# Racial Differences in the Molecular Genetic Biomarkers of Diffuse Large B-Cell Lymphoma

**DOI:** 10.3390/biomedicines13112782

**Published:** 2025-11-14

**Authors:** Marco D. Gomes, Kevin Sun, Ji Li, William Middlezong, Victoria Stinnett, Laura Morsberger, Ying S. Zou, Yi Huang

**Affiliations:** 1 Department of Pathology, Johns Hopkins University School of Medicine, Baltimore, MD 21287, USA; marcoutsw2020@gmail.com (M.D.G.); kevinsuperhy@gmail.com (K.S.); wmiddle2@alumni.jh.edu (W.M.); vlloyd3@jhmi.edu (V.S.); lmorsber@jhmi.edu (L.M.); 2 Department of Mathematics and Statistics, University of Maryland Baltimore County, Baltimore, MD 21250, USA; jil1@umbc.edu (J.L.); yihuang@umbc.edu (Y.H.)

**Keywords:** DLBCL, racial disparities, gene rearrangements, *MYC*, *BCL2*, *IGH*::*BCL2*, FISH, gene interactions, HGBCL, molecular biomarkers

## Abstract

**Background/Objectives:** Diffuse large B-cell lymphoma (DLBCL) exhibits pronounced racial disparities in incidence and outcomes, yet the molecular basis remains poorly understood. Here, we examined racial differences in gene rearrangements (*MYC*, *BCL2*, *BCL6*), fusions (*IGH*::*MYC*, *IGH*::*BCL2*), and their interactions among White, Black, Asian, and Other-race groups in patients with DLBCL to uncover genetic drivers of disparities. **Methods:** We analyzed 919 DLBCL cases (2006–2023) from Johns Hopkins Hospital using fluorescence in situ hybridization to detect gene abnormalities. We used logistic regression and proportional odds models, adjusted for age and sex, to evaluate racial differences in five gene abnormalities and 10 gene–gene interaction pairs. Pearson’s Chi-squared and Goodman–Kruskal’s gamma tests assessed prevalence and interaction severity across racial groups. **Results:** *MYC* rearrangements and the MYC*IGH::*MYC* interaction were marginally more frequent in the White group than in Black and Other groups (*p* = 0.092, *p* = 0.098, respectively). *IGH*::*BCL2* fusions were more prevalent in the Asian group than in the White group (*p* = 0.095), and the BCL2*IGH::*BCL2* interaction was significantly higher in the Asian group (*p* = 0.049) than in the White group. Although high-grade B-cell lymphoma (HGBCL) prevalence showed no significant racial differences (*p* = 0.16), the Asian group exhibited a higher proportion of aggressive HGBCL with concurrent *IGH*::*MYC* and *IGH*::*BCL2* fusions compared with the White group (*p* = 0.076). Age significantly influenced all gene abnormalities and interactions (*p* < 0.001–0.052), except for *MYC* rearrangements and specific pairs. Sex and sex–race interactions showed no significant effects. **Conclusions:** This study highlights molecular contributions to the racial differences in DLBCL disease. Further research collecting ancestry-specific biomarkers, treatment regimens, and clinical variables and outcomes is needed to advance personalized treatment strategies.

## 1. Introduction

Lymphoma, a heterogeneous group of hematologic malignancies originating from lymphoid tissues, represents a significant global health challenge owing to its diverse clinical presentations and complex molecular mechanisms [1,2,3,4,5]. Encompassing both Hodgkin and non-Hodgkin subtypes, lymphomas account for approximately 3% to 5% of all cancers worldwide, with incidence rates rising in recent decades [2,3,4,5]. Diffuse large B-cell lymphoma (DLBCL), the most prevalent subtype of non-Hodgkin lymphoma, represents approximately 30% of adult lymphoma cases worldwide [4,5,6,7]. DLBCL is an aggressive disease, and untreated patients face a median survival of <1 year [8]. DLBCL outcome is heterogeneous, with some patients experiencing inferior outcomes despite therapeutic advances [9]. Biologic, clinical, and environmental factors contribute to this heterogeneity, and well-documented racial disparities in DLBCL significantly affect disease presentation, treatment access, and survival outcomes [10,11,12].

The incidence of DLBCL is higher in White individuals than in other racial groups [13,14,15]. White patients are typically diagnosed with DLBCL at older ages, whereas Black patients often present with more advanced disease and B symptoms, such as fever, night sweats, and weight loss [16]. Additionally, Black patients experience lower 1-year relative survival, 5-year disease-specific survival, and overall survival rates than White patients do [16]. Racial differences in DLBCL survival vary according to stage. For stage I disease, White patients have the best 5-year survival rate, whereas Black patients have the worst. Asian/Pacific Islanders have the worst survival rate for stage IV disease [13]. Although patients with DLBCL have experienced improvements in 5-year survival rates, persistently lower survival rates have been reported among patients in racial/ethnic minority groups, such as Asian and Black patients, when compared with White patients [16,17,18]. However, one study suggests that White patients may experience higher mortality risk than certain minority groups, potentially because of differences in disease etiology, independent of clinical prognostic factors and treatment approaches [19]. Socioeconomic factors, including insurance status and income, often contribute to these disparities, with minority patients more likely to be uninsured or in lower income brackets or to face barriers to healthcare access [11,15]. For example, Black patients are less likely than White patients to receive autologous hematopoietic cell transplantation and chemotherapy [16,20].

Genetic factors, such as gene rearrangements, fusions, mutations, copy number alterations, and epigenetic change, play a central role in the pathogenesis of DLBCL [21,22]. Key rearrangements involving *MYC*, *BCL2*, and *BCL6*, alongside mutations in genes such as *MYD88*, *EZH2*, *TP53*, *KMT2D*, *CREBBP*, *NOTCH1*/*2*, and *B2M*, drive lymphomagenesis by promoting proliferation, inhibiting apoptosis, blocking differentiation, or enabling immune evasion. The *BCL2*, *BCL6*, and *MYC* genes have important proto-oncogenic roles [23,24,25]. *BCL2* encodes an anti-apoptotic protein, *BCL6* encodes a transcriptional repressor that regulates B-cell differentiation and germinal center formation critical for normal immune responses, and *MYC* encodes a transcription factor that regulates cell proliferation, metabolism, and apoptosis of numerous genes involved in cell growth. In DLBCL, *MYC* rearrangements typically involve the *MYC* gene translocating with immunoglobulin genes, such as immunoglobulin heavy chain (*IGH*), or immunoglobulin light chains (lambda, immunoglobulin light lambda chain and kappa, immunoglobulin light kappa chain), as well as non-immunoglobulin (non-IG) partner genes [26,27]. These translocations induce *MYC* overexpression. Overexpression of *MYC*, *BCL2*, and *BCL6* genes plays a critical role in the pathogenesis of DLBCL, with reported frequencies ranging from 8% to 40% of DLBCL cases [28,29].

Recent genomic advancements have identified distinct molecular subtypes that contribute to variability in prognosis and response to therapies [22]. Concurrent rearrangements of *MYC* and *BCL2* are hallmark alterations in aggressive B-cell lymphomas, also known as double-hit lymphomas, which are classified as high-grade B-cell lymphomas (HGBCLs) [22,29,30,31,32]. HGBCLs are primarily diagnosed through fluorescence in situ hybridization (FISH), the gold standard for detecting concurrent *MYC* (8q24) and *BCL2* (18q21) gene rearrangements, including *IGH::MYC* and *IGH::BCL2* gene fusions [2,26]. Immunohistochemistry complements FISH by identifying high MYC and BCL2 protein expression, raising suspicion for HGBCLs and guiding further testing. These genetic alterations drive rapid cell proliferation (MYC) and inhibit apoptosis (BCL2), contributing to HGBCL’s highly aggressive behavior and poor prognosis with standard therapies [33,34]. HGBCL often exhibits resistance to standard-of-care R-CHOP (rituximab with cyclophosphamide, doxorubicin, vincristine, and prednisone) therapy and is associated with shorter median survival [2,28,33,34]. Intensive regimens, such as DA-EPOCH-R, and targeted therapies, like venetoclax for BCL2, are frequently used to improve outcomes in these challenging cases [35,36,37].

Although genetic factors are critical in DLBCL pathogenesis, the effect of race on these factors remains underexplored. Prior studies indicate that mutations in key DLBCL driver genes (*ATM*, *MGA*, *SETD2*, *TET2*, *MLL3*, and *DNMT3A*) are more frequent in Black patients than in White patients [38]. Additionally, *BCL6* gene alterations are less frequent in Taiwanese patients with DLBCL than in Western populations [39] but are more prevalent in Chinese patients with germinal center B-cell-like DLBCL than in American patients [40]. However, it is unclear how gene rearrangements or fusions involving *BCL2* and *MYC* (key hallmarks of HGBCL), *BCL6* rearrangements, and their interaction with racial backgrounds drive DLBCL pathogenesis and prognosis. In this study, we examined the interplay between racial backgrounds and gene rearrangements or fusions involving *BCL2*, *BCL6*, and *MYC* in patients with DLBCL to enhance understanding of their roles in disease pathogenesis and clinical outcomes, with the goal of advancing personalized diagnostic and therapeutic approaches for improved outcomes.

## 2. Materials and Methods

### 2.1. Study Population

We conducted a clinical database query for lymphoma cases (n = 2136) analyzed by the Johns Hopkins Hospital (JHH) lymphoma FISH panel as part of routine lymphoma testing from 7 August 2006 to 1 May 2023. From these specimens, we identified a study cohort of 1167 cases with a confirmed diagnosis of DLBCL. Patients with non-B-cell lymphomas and other B-cell lymphoma subtypes, as well as those who did not self-report race, were excluded from this study. We classified disease cases based on World Health Organization criteria using clinical, morphologic, immunophenotypic, cytogenetic, and molecular genetic features [2,3]. The final analytic sample included 919 patients with DLBCL patients (Supplemental Table S1). 248 patients were excluded due to missing race data. Patients’ characteristics and the frequency of genetic alternations were compared between the analytic cohort (n = 919) and the excluded group (n = 248; Supplemental Table S2). The two groups were well balanced, with no significant differences in any variable except *BCL2* alteration frequency.

### 2.2. FISH for Gene Biomarkers

**Figure 1:**
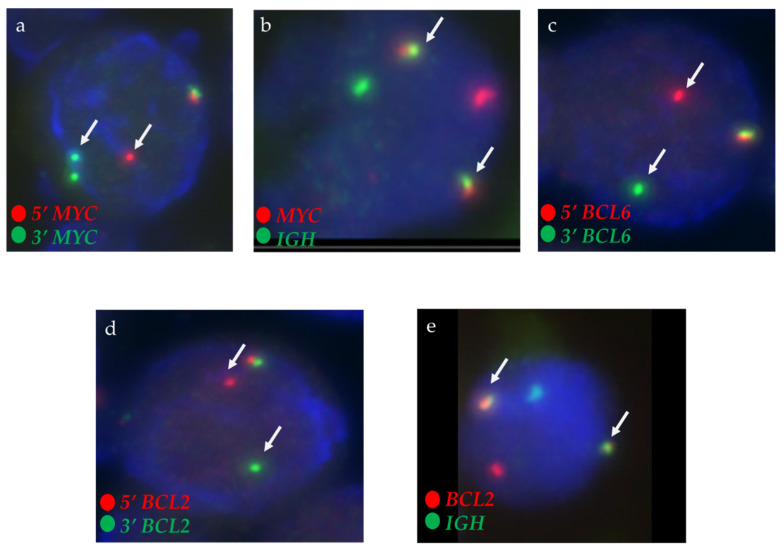
FISH on formalin-fixed paraffin-embedded tumor specimen. (**a**) *MYC* break-apart FISH assay revealing characteristic *MYC* gene rearrangement, indicated by separation (split) of red and green signals (arrows). (**b**) *IGH*::*MYC* dual-fusion FISH assay demonstrating *IGH::MYC* fusion, as evidenced by colocalized red and green signals forming a yellow signal (arrows). (**c**) *BCL6* break-apart FISH assay revealing characteristic *BCL6* gene rearrangement, indicated by separation (split) of red and green signals (arrows). (**d**) *BCL2* break-apart FISH assay revealing characteristic *BCL2* gene rearrangement, indicated by separation (split) of red and green signals (arrows). (**e**) *IGH*::*BCL2* dual-fusion FISH assay demonstrating *IGH::BCL2* fusion, as evidenced by colocalized red and green signals forming a yellow signal (arrows).

The JHH standard B-cell lymphoma FISH panels, performed on formalin-fixed paraffin-embedded tumor specimens, targeted *MYC* [8q24], *BCL2* [18q21], *BCL6* [3q27], and *IGH* [14q32] to detect rearrangements and specific fusions, including *IGH*::*MYC* [t(8;14)(q24;q32)] and *IGH*::*BCL2* [t(14;18)(q32;q21)], in B-cell lymphoma cases. We used break-apart probes to identify rearrangements of *MYC*, *BCL2*, and *BCL6*, and dual-fusion probes to confirm *IGH*::*MYC* and *IGH*::*BCL2* translocations (Abbott Molecular, Inc., Des Plaines, IL, USA), following the manufacturer’s protocol as previously described [41]. Two technologists independently evaluated 100 interphase nuclei per probe using fluorescence microscopy on a Zeiss Axioscope system. Consistent with established laboratory thresholds and clinical guidelines, we defined a positive rearrangement for break-apart probes as ≥10% of nuclei displaying abnormal (e.g., split) signals and defined a positive gene fusion for dual-fusion probes as ≥15% of nuclei showing abnormal (e.g., fusion) signals (Figure 1).

### 2.3. Gene Rearrangement and Gene Fusion in Lymphoma

Each of the five gene abnormalities, *MYC*, *BCL2*, *BCL6*, *IGH::MYC*, and *IGH::BCL2*, were assessed as a binary outcome (0 = normal, 1 = abnormal) based on FISH results. For the five genes of interest, we examined all possible gene–gene interaction pairs. For each of the 10 pairs, the combined binary status of the two genes yielded four possible abnormality patterns: (0,0), (0,1), (1,0), and (1,1). To improve statistical power for detecting racial differences across these gene–gene interaction patterns, we redefined the “gene-interaction-pair” outcome as an ordinal categorical variable with three levels (0, 1, and 2), reflecting the severity of abnormalities. Specifically: a gene-pair value of **0** indicated that both genes were normal (0,0), representing no rearrangements or fusions and the lowest severity; **1** indicated that one out of the two genes was abnormal (0,1) or (1,0), representing moderate severity with a single rearrangement or fusion; and **2** indicated that both genes were abnormal (1,1), representing the highest severity with dual abnormalities, often linked to more aggressive disease profiles—as exemplified by double-hit lymphoma—and portend inferior outcomes with standard therapies [22,29,30,31,32]. This approach provided a more meaningful and statistically efficient alternative to treating the outcome as a nominal four-category variable.

Because HGBCL is highly aggressive and associated with a poor prognosis and shorter median survival, we defined patients as having HGBCL if they had at least one abnormality in *MYC* or *IGH*::*MYC* and at least one abnormality in *BCL2* or *IGH*::*BCL2*.

### 2.4. Covariates and Potential Confounders

Our data initially classified race into four categories: White (race = 0), Black (race = 1), Asian (race = 2), and Other (race = 3). The “Other” group included mixed or less-defined racial/ethnic categories, such as American Indian, Alaskan native, etc. Because the “Other” sample sizes were much smaller than that of the Black group, we combined race into two and three groups to improve statistical power for detecting racial differences in gene abnormalities. The two-group race variable was White (race = 0) and non-White (race = 1, 2, 3), where the non-White group comprised Black, Asian, and Other; Black patients represented ~61% (157 of 258) of the non-White group. To determine how the Asian population differs from the White group, we used the three-group race variable, which included White (race = 0), Black and Other (race = 1, 3), and Asian (race = 2). In that scenario, Black patients accounted for ~78% (157 of 201) of the Black and Other group. Age and sex were adjusted as potential confounders.

### 2.5. Statistical Analysis

To assess the racial differences in molecular genetic biomarkers (five individual gene abnormalities) and the association between race and sex among patients with B-cell lymphoma, we used Pearson’s chi-square tests. For racial differences in age, both *t* test and *F* test were used for two versions of race variables. We evaluated racial differences in 10 gene–gene interaction pairs using Goodman–Kruskal’s gamma test, which offers greater power than chi-square tests by accounting for the ordinal nature of gene abnormality severity levels. We visualized the prevalence of gene abnormalities across racial groups using bar charts.

To confirm the racial differences in the genetic biomarkers with confounding adjustments, including individual gene abnormality and gene–gene interaction pairs, we used different types of logistic regressions. For each of the five gene abnormalities, we used binary logistic regression. For gene–gene interaction pairs (three severity levels: both normal, one abnormal, both abnormal), we applied proportional odds models, also known as the ordered logistic regression, which more powerfully detected racial differences in the gene–gene interactions than multinomial logistic models did (ignoring the ordering of gene abnormality severity levels). Sequential model building was used to refine the regressions, including adjustments for potential confounders and pairwise interactions (sex, race, and age). Although analyses were initially performed using four race categories, the Black and Other groups showed no statistically significant differences across gene abnormalities, gene–gene interaction levels, or HGBCL status (Supplemental Tables S3 and S4). Therefore, these two groups were combined to improve model parsimony and estimation efficiency without altering the substantive conclusions. We selected the final model based on AIC, BIC, and likelihood ratio tests. Sex and interaction terms were not statistically significant in any model and were excluded. Final models included three-category race (White, Asian, Black and Other) and age as covariates. Statistical significance was defined as *p* < 0.05 (two-sided) for all hypothesis tests reported in this study. All analyses were conducted using R 4.4.3 (RStudio 2021).

## 3. Results

### 3.1. Patient Characteristics and Racial Differences in Gene Abnormality Prevalence

Among the 919 patients with lymphoma, the White group was the largest, with 661 individuals (~72% of the sample), and the non-White group comprised 258 individuals (~28%; Table 1). Within the non-White group, the Black subgroup was the largest (157 individuals), followed by Asian (57) and Other (44). The average age of the entire sample was 63.6 years (standard deviation [SD] = 17.11). The statistically significant difference in age between White and non-White groups (*p* = 0.005) suggested that the White patients were, on average, significantly older than the non-White patients, in particular the Black and Other group, consistent with published data [13,38]. The proportions of male and female were relatively consistent across racial groups, with no significant differences between White and non-White groups. No significant differences in disease types or cell-of-origin subtypes were observed across the four racial groups or the specific pairwise comparisons (e.g., Black & Others vs. White, Asian vs. White, White vs. non-White patients) (Supplemental Table S1).

**Figure 2:**
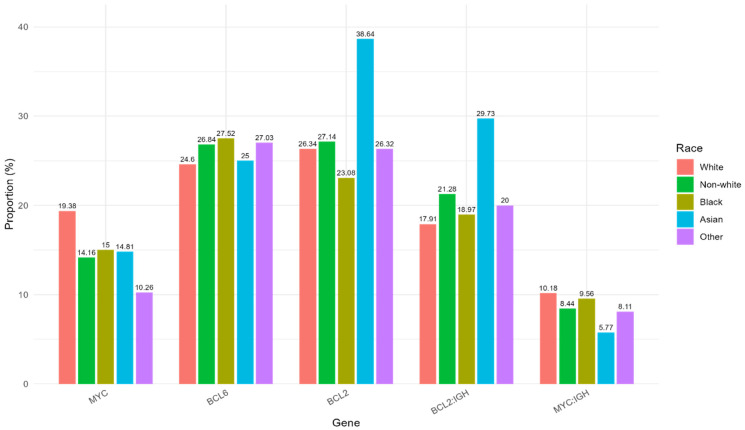
The prevalence of specific gene abnormalities detected by FISH and stratified by race. *X*-axis shows genes including *MYC*, *BCL6*, *BCL2*, *IGH*::*BCL2*, and *IGH*::*MYC*. *Y*-axis shows the proportions of gene abnormalities across racial groups: White, Non-White (comprising Black, Asian, and Other), Black, Asian, and Other patients.

Table 1 and Figure 2 show the racial differences in the prevalence of five different genetic subtypes, including rearrangements of *MYC*, *BCL2*, and *BCL6* genes and gene fusions of *IGH*::*MYC* and *IGH*::*BCL2*. The prevalence of *MYC* rearrangement was higher in the White group (19.38%) than in the non-White group (14.16%), but this difference was only marginally statistically significant (0.05 < *p* = 0.098 < 0.10 threshold). Although there were no significant differences in the prevalence of *BCL2* and *BCL6* gene rearrangements and *IGH*::*MYC* and *IGH*::*BCL2* gene fusions across the White and non-White groups (Table 1), the Asian group seemed to have a higher prevalence of *BCL2* gene rearrangement (38.64%) and *IGH*::*BCL2* gene fusion (29.73%) (Figure 2).

### 3.2. Racial Differences in Gene Abnormalities with Adjustments for Confounders

Table 2 shows the results on the racial differences in gene abnormalities among Black and Other, Asian, and White groups, after we adjusted for confounders using logistic regression.

*MYC* and *IGH*::*BCL2* exhibited marginally significant racial differences (0.05 < *p* < 0.1): the odds of having *MYC* rearrangements in the Black and Other group were 0.66 times the odds in the White group (*p* = 0.092), with 95% confidence intervals (CI) (0.41, 1.05), which means the White group had a higher proportion of *MYC* rearrangements than the Black and Other group did. In addition, the odds of having *IGH*::*BCL2* fusions in the Asian group were 1.90 times the odds in the White group (*p* = 0.095), with 95% CI (0.86, 3.96), which means that the Asian group had a higher proportion of *IGH*::*BCL2* gene fusions than the White group did, after adjusting for confounders. Remark that even though marginally significant racial differences in *MYC* and *IGH*::*BCL2* abnormality were observed, these findings were exploratory and needed future studies to confirm this association. No significant racial differences were observed for other gene abnormalities (*p* = 0.112 to ~0.998), suggesting that race did not strongly influence the prevalence of these gene abnormalities in this study. Age was statistically significantly associated with *BCL6* and *BCL2* rearrangements and *IGH*::*MYC* and *IGH*::*BCL2* fusions (*p* < 0.001 to ~0.047), but not with *MYC* rearrangements. The older the patients were, the more likely they were to have the rearrangements of *BCL6* and *BCL2* genes and *IGH*::*BCL2* gene fusion; however, older patients were less likely to have *IGH*::*MYC* gene fusion. Neither sex nor the sex–race interaction significantly affected any gene abnormalities.

### 3.3. Racial Differences in the Gene–Gene Interaction Pairs and HGBCL

**Figure 3:**
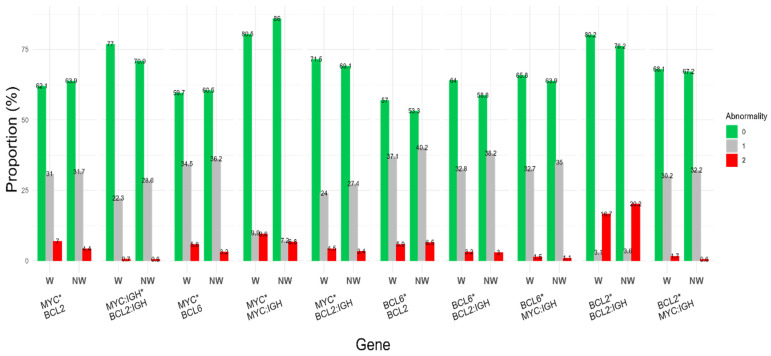
Race differences in 10 gene–gene interaction pairs. The bar chart shows the proportion of three abnormality severity levels for 10 gene–gene interaction pairs (gene1*gene2) in two race groups, White (W) and non-White (NW). Severity was determined by the number of abnormal genes in each pair (0, both genes are normal; 1, one of the genes is abnormal; 2, both genes are abnormal). Numeric details are available in Supplemental Table S5.

Figure 3 and Supplemental Table S5 show the racial differences in abnormality severity level of 10 gene–gene interaction pairs between White and non-White groups. The gene–gene interactions involved combinations of genetic biomarkers (*MYC*, *BCL2*, *BCL6*, *IGH*::*MYC*, *IGH*::*BCL2*) related to lymphoid malignancies. The abnormality prevalence of the MYC*IGH::*MYC* gene pair in the White group was statistically significantly higher than that in the non-White group (*p* = 0.05), consistent with *MYC* results shown in Figure 2. Although the non-White group showed higher abnormality prevalences in the *IGH*::MYC*IGH::*BCL2* pair, BCL2*IGH::*BCL2* pair, and BCL6*IGH::*BCL2* pair than the White group did, none of the differences were statistically significant (*p* = 0.13 to 0.29). No other gene interaction pairs showed significant differences, suggesting that race did not strongly influence the abnormality prevalences of these gene–gene interactions in this study.

Of 619 patients with available *MYC*, *IGH*::*MYC, BCL2*, and *IGH*::*BCL2* data in this study, 45 patients with HGBCL (7.3%) were identified (Table 3). Although both the White (8.30%) and Asian (8.11%) groups had higher prevalences of aggressive HGBCL than the Black (3.81%) and Other (3.23%) groups did, this racial difference was not statistically significant based on the *p* value from the chi-square test (*p* = 0.16). However, the observed count of patients with HGBCL in the Asian and Other groups were low, which might underpower the statistical test, suggesting future large studies are needed for further investigation.

### 3.4. Racial Differences in the Gene–Gene Interaction Pairs and HGBCL After Adjustment for Confounders

We analyzed race differences in gene–gene interaction abnormality severity levels among Black and Other, Asian, and White groups and adjusted for confounders with the proportional odds model (Table 4). Table 4 strengthened our findings from Figure 2 and found that the BCL2*IGH::*BCL2* gene pair exhibited a significant racial difference. Specifically, the odds of having *IGH*::*BCL2* fusion among *BCL2* rearrangements in the Asian group was 2.11 (95% CI: 1.00, 4.38) times the odds in the White group (*p* = 0.049) after we adjusted for confounders. In other words, Asian patients exhibited a much higher proportion of *IGH*::*BCL2* fusion among *BCL2* rearrangements than White patients did. The MYC*IGH::*MYC*, BCL6*IGH::*BCL2*, and *IGH*::MYC*IGH::*BCL2* gene pairs exhibited marginally significant racial differences (0.05 < *p* < 0.1). The White group had a higher proportion of *IGH*::*MYC* fusion among *MYC* rearrangements than the Black and Other group did (odds ratio = 1.49, 95% CI (0.94, 2.50), *p* = 0.098), after adjusting for confounding. Additionally, the Asian group had a higher proportion of *IGH*::*BCL2* fusion among *BCL6* rearrangements (odds ratio = 1.78, 95% CI (0.90, 3.51), *p* = 0.096) and the concurrent *IGH*::*MYC* and *IGH*::*BCL2* fusions (odds ratio = 1.92, 95% CI (0.91, 3.89), *p* = 0.076) than the White group did. Remark that even though marginally significant racial differences in the above three gene pairs were observed, these findings were exploratory, suggesting future studies to confirm them. Other gene–gene interaction pairs showed no significant racial differences. Age was significantly associated with most gene–gene interaction pairs (*p* < 0.001 to 0.052) but not with MYC*IGH::*MYC*, MYC*BCL6, and BCL6*IGH::*MYC*. Thus, with advancing age, patients were more likely to have a higher gene–gene interaction abnormality severity level. Although aggressive HGBCL did not show significant racial differences, older age was confirmed as a significant risk factor for increasing risk of aggressive HGBCL. Neither sex nor the sex–race interaction significantly affected any gene–gene interaction pairs or HGBCL.

## 4. Discussion

This study represents one of the largest examinations to date of racial differences in key molecular genetic biomarkers of DLBCL, specifically focusing on rearrangements in *MYC*, *BCL2*, and *BCL6* genes and on gene fusions involving *IGH*::*MYC* and *IGH*::*BCL2* and their interactions. By analyzing FISH data from 919 DLBCL patients at a single institution over a 17-year period, we identified subtle but noteworthy racial differences in these biomarkers that may contribute to the well-documented disparities in DLBCL incidence, clinical presentation, and survival outcomes [10,11,12,13,14,15]. Our results suggest possible distinct racial patterns in *MYC* rearrangements, *IGH*::*BCL2* fusions, and specific gene–gene interactions associated with aggressive HGBCL after adjusting for age and sex. These findings underscore the potential role of ancestry-specific genetic alterations in driving DLBCL heterogeneity and emphasize the need for tailored approaches in molecular diagnostics and therapy.

The group consisting of White patients exhibited a marginally higher prevalence of *MYC* rearrangements (19.38% vs. 14.16% in non-White patients, *p* = 0.092). This finding indicates a greater burden of proliferation-driving genetic alterations in White patients, potentially contributing to the higher incidence of DLBCL in this population [13]. Gene–gene interactions revealed that the MYC*IGH::*MYC* interaction was also significant (*p* = 0.05), with White patients showing more dual alterations. The presence of *MYC* rearrangements, particularly *IGH*::*MYC* fusions*,* may also be associated with more aggressive disease phenotypes and worse prognoses in patients treated with standard therapy, as supported by prior studies [33,34,42,43,44,45].

Conversely, the group consisting of Asian patients showed a slightly higher prevalence of *IGH*::*BCL2* fusions (*p* = 0.095) when compared with the White group. These fusions, frequently driven by the t(14;18)(q32;q21) translocation, promote anti-apoptotic signaling through *BCL2* overexpression under *IGH* enhancer control. Gene–gene interaction analysis revealed a significant BCL2*IGH::*BCL2* interaction (*p* = 0.049), with the Asian group displaying a higher proportion of *IGH*::*BCL2* fusions among those with *BCL2* rearrangements. This finding implies a stronger linkage between *BCL2* disruption and *IGH*-mediated translocations in Asian individuals, potentially leading to more aggressive disease phenotypes. It may partially explain reports of lower survival rates in Asian populations with advanced-stage DLBCL [13]. No significant racial variations were observed for *BCL6* or *BCL2* rearrangements alone, indicating that racial differences may be more pronounced in specific oncogenic pathways rather than broadly across all biomarkers.

Although overall HGBCL prevalence showed no significant racial differences (*p* = 0.16), the Asian group showed a marginally higher proportion of aggressive HGBCL with concurrent *IGH*::*MYC* and *IGH*::*BCL2* fusions (*p* = 0.076 compared with the White group), a hallmark of double-hit lymphomas known for treatment resistance [29,30,31,32]. These interactions are clinically relevant, as concurrent *MYC* and *BCL2* abnormalities define HGBCL, a subtype with poor prognosis under standard R-CHOP therapy [34,43]. The elevated frequency of dual *IGH*::*MYC* and *IGH*::*BCL2* fusions in the Asian group (4.9% of the cohort had HGBCL) suggests an enrichment of double-hit lymphomas, potentially contributing to the worse outcomes reported in this population [13]. The *IGH* locus is a frequent translocation partner for *MYC* and *BCL2* genes in B-cell lymphomas, driving oncogenic overexpression that promotes uncontrolled proliferation (MYC) and inhibits apoptosis (BCL2). These *IGH*-mediated translocations are critical drivers of aggressive lymphomas and correlate with inferior median overall survival compared with non-IG partners owing to enhanced oncogene expression [28].

Age emerged as a significant confounder across most biomarkers and interactions (*p* < 0.001 to 0.052), except for *MYC* rearrangements and certain pairs like MYC*BCL6. Increasing age was associated with higher odds of *BCL2* and *BCL6* rearrangements, *IGH*::*BCL2* fusions, and elevated gene-pair interaction levels. This finding aligns with the known epidemiology of DLBCL, in which incidence increases with age, and older patients often present with more complex genetic profiles, potentially due to cumulative genetic instability over time- or age-related oncogenic pathways involving epigenetic changes or mutations in genes such as *CREBBP*, *EZH2*, and *KMT2D* [22]. Notably, no significant sex or sex–race interaction effects were observed, indicating that these molecular differences are not strongly influenced by sex, in contrast to some clinical reports of sex-based differences in treatment response [16]. Our cohort’s demographic profile, with the White group comprising 72% and being older on average (*p* = 0.005), aligns with U.S. lymphoma trends, in which the White populations have a higher incidence but Black populations tend to present with disease at younger ages [16].

These molecular findings should be interpreted in the context of broader health disparities. Racial differences most likely arise from a complex interplay of genetic ancestry, environmental exposures, and socioeconomic factors. Genome-wide studies have linked African ancestry to distinct mutations (e.g., *ATM*, *TET2*) and worse outcomes [38], but East Asian patients exhibit unique *BCL6* alteration patterns, potentially because of germline variants or immune-related mechanisms [21,22,39,40]. Socioeconomic barriers, including limited access to care, inadequate insurance coverage, and lower income, disproportionately affect minority groups, making access to advanced therapies like autologous hematopoietic cell transplantation difficult [16,20]. Such societal factors may amplify the molecular differences by delaying diagnosis or treatment, though adjustments for socioeconomic status partially mitigate this gap, underscoring the interplay of ancestry, environment, and healthcare access. However, our single-institution cohort, despite its size, may not fully reflect these dynamics, as it primarily represents an urban, underserved population [17,19].

Despite its strengths, including a large sample size (n = 919) and rigorous FISH-based detection, this study has limitations. Self-reported race may not reflect genetic ancestry, risking misclassification bias. The heterogeneous “Other” category and small subgroup sizes limited statistical power, necessitating combined non-White analyses that may obscure subgroup-specific effects. The focus on FISH-detectable rearrangements and fusions excluded key molecular alterations like mutations (e.g., *MYD88*, *EZH2*, *TP53*), copy number variations, and epigenetic changes critical to lymphomagenesis. Multi-omics data (e.g., transcriptomics, proteomics) and gene–environment interactions (e.g., lifestyle, exposures) were not explored. Genomic databases, biased toward European ancestries, may miss minority-specific variants. Additional limitations include the single-institution JHH cohort, which may limit national generalizability, and the absence of clinical outcome data, precluding linkages between biomarkers and survival disparities. Key patient variables—such as comorbidities, treatment regimens, and performance status—were unavailable, further constraining multivariable adjustments. Given the exploratory nature of our study and these data gaps, our findings on racial differences in genetic biomarkers should be interpreted cautiously. Future multicenter studies incorporating comprehensive clinical, therapeutic, and longitudinal outcome data are warranted to validate these observations. The retrospective design and reliance on routine clinical FISH data introduced selection bias. Although the 248 patients excluded for missing race data were balanced with the analytic cohort on all baseline variables except *BCL2* alteration (as shown in Table S2), suggesting minimal selection bias and negligible confounding of primary findings, this exclusion reduced statistical power for race-stratified analyses. Small sample sizes for rare events like HGBCL (45 cases) further limited power, potentially causing underestimations of racial differences. The absence of additional molecular data, such as mutations or immunohistochemistry, restricted refined HGBCL classification [2,29]. Despite our study’s alignment with broader epidemiologic trends [13,14,15], these limitations warrant cautious interpretation.

Future research should incorporate ancestry-informative markers, larger multi-ethnic cohorts and integrated omics approaches to dissect biologic versus socioeconomic contributions. Longitudinal studies that track treatment regimens, responses, and clinical data/outcomes (e.g., survival, stage) in racially diverse groups could validate these biomarkers for precision medicine. Addressing healthcare inequities through targeted interventions may also mitigate disparities. By recognizing these molecular racial differences, this study lays the groundwork for precision medicine approaches to improve DLBCL outcomes across diverse populations.

## 5. Conclusions

This study provides novel insights into racial disparities in molecular genetic biomarkers of DLBCL, identifying subtle but meaningful differences in *MYC* rearrangements, *IGH*::*BCL2* fusions, and specific gene interactions among White, Black, Asian, and Other racial groups. Notably, White patients exhibited a marginally higher prevalence of *MYC* rearrangements, particularly *IGH*::*MYC* fusion, whereas Asian patients showed increased *IGH*::*BCL2* fusions and stronger *IGH*::BCL2*IGH::*MYC* interactions, with a trend toward more aggressive HGBCL. These findings suggest that ancestry-specific genetic alterations may contribute to the heterogeneity in DLBCL presentation and outcomes. Despite limitations such as smaller minority sample sizes and retrospective design, our results underscore the importance of integrating a patient’s molecular profile with racial and socioeconomic factors to advance personalized diagnostics and therapies. Future multi-institutional studies with comprehensive genomic analyses are essential to validate these findings and develop targeted interventions to reduce disparities and improve outcomes for patients with DLBCL.

## Figures and Tables

**Table 1:** Race differences in genetic biomarkers, age, and sex.

Variable ^a^	Overall	White	Non-White	*p* Value	Black	Asian	Other
	(n = 919)	(n = 661)	(n = 258)		(n = 157)	(n = 57)	(n = 44)
Age, mean (SD)	63.58 (17.11)	64.59 (16.78)	61.00 (17.69)	**0.005**	61.25 (16.38)	65.6 (16.74)	54.14 (21.37)
Sex, female	369 (40.15)	258 (39.03)	111 (43.02)	0.3	69 (43.95)	25 (43.86)	17 (38.64)
*MYC*	145 (17.88)	112 (**19.38)**	33 (14.16)	**0.098**	21 (15)	8 (14.81)	4 (10.26)
*BCL6*	173 (25.22)	122 (24.60)	51 (26.84)	0.61	30 (27.52)	11 (25)	10 (27.03)
*BCL2*	187 (26.56)	133 (26.34)	54 (27.14)	0.90	27 (23.08)	17 **(38.64)**	10 (26.32)
*IGH*::*BCL2*	124 (18.87)	84 (17.91)	40 (21.28)	0.38	22 (18.97)	11 **(29.73)**	7 (20)
*IGH*::*MYC*	77 (9.69)	58 (**10.18**)	19 (8.44)	0.54	13 (9.56)	3 (5.77)	3 (8.11)

^a^ All values except for age are presented as n (%). SD = standard deviation. Bold font shows either statistically significant or marginal statistically significant (0.05 < *p* < 0.10).

**Table 2:** Race differences in gene abnormalities with adjustments for confounders.

	Logistic Regression	
Variable	Odds Ratio (95% CI)	*p* Value
*MYC*		
Race: Black & Other vs. White	**0.66 (0.41, 1.05)**	**0.092**
Race: Asian vs. White	0.73 (0.31, 1.50)	0.420
Age	1.0 (0.99, 1.01)	0.613
*BCL6*		
Race: Black & Other vs. White	1.23 (0.80, 1.87)	0.331
Race: Asian vs. White	1.00 (0.47, 1.99)	0.998
Age	**1.01 (1.00, 1.02)**	**0.047**
*BCL2*		
Race: Black & Other vs. White	0.99 (0.64, 1.51)	0.954
Race: Asian vs. White	1.69 (0.87, 3.21)	0.112
Age	**1.03 (1.01, 1.04)**	**<0.001**
*IGH*::*BCL2*		
Race: Black & Other vs. White	1.24 (0.76, 1.99)	0.371
Race: Asian vs. White	**1.90 (0.86, 3.96)**	**0.095**
Age	**1.03 (1.01, 1.04)**	**<0.001**
*IGH*::*MYC*		
Race: Black & Other vs. White	0.81 (0.44, 1.43)	0.489
Race: Asian vs. White	0.55 (0.13, 1.58)	0.336
Age	**0.98 (0.97, 1.00)**	**0.006**

CI = confidence interval. Bold font shows either statistically significant or marginal statistically significant (0.05 < *p* < 0.10).

**Table 3:** Race differences in genetic biomarkers in HGBCL.

HGBCL ^a^	White	Non-White	Black	Asian	Other	*p* Value
HGBCL = 1 (Aggressive type, 45 patients)	37 (8.30)	8 (4.62)	4 (3.81)	3 (8.11)	1 (3.23)	0.16
HGBCL = 0 (Non-aggressive type, 574 patients)	409 (91.70)	165 (95.38)	101 (96.19)	34 (91.89)	30 (96.77)	

Data are shown as n (%). ^a^ HGBCL was defined as at least one of (*MYC*, *IGH*:: *MYC*) = 1 (abnormal), AND at least one of (*BCL2*, *IGH*::*BCL2*) = 1 (abnormal).

**Table 4:** Racial differences in gene–gene interaction abnormality severity levels and HGBCL with adjustments for confounders.

	Proportional Odds Model	
Variable	Regression Coefficient (95% CI)	*p* Value
MYC*IGH::*MYC*		
Race: Black & Other vs. White	**0.67 (0.40, 1.06)**	**0.098**
Race: Asian vs. White	0.64 (0.26, 1.37)	0.292
Age	1.00 (0.99, 1.01)	0.432
*IGH*::MYC*IGH::*BCL2*		
Race: Black & Other vs. White	1.33 (0.85, 2.06)	0.204
Race: Asian vs. White	**1.92 (0.91, 3.89)**	**0.076**
Age	**1.01 (1.00, 1.02)**	**0.052**
BCL2*IGH::*BCL2*		
Race: Black & Other vs. White	1.24 (0.75, 2.01)	0.387
Race: Asian vs. White	**2.11 (1.00, 4.38)**	**0.049**
Age	**1.03 (1.01, 1.04)**	**<0.001**
BCL6*IGH::*BCL2*		
Race: Black & Other vs. White	1.27 (0.84, 1.93)	0.255
Race: Asian vs. White	**1.78 (0.90, 3.51)**	**0.096**
Age	**1.02 (1.01, 1.03)**	**<0.001**
MYC*BCL2		
Race: Black & Other vs. White	0.84 (0.56, 1.24)	0.386
Race: Asian vs. White	1.33 (0.70, 2.46)	0.377
Age	**1.01 (1.01, 1.02)**	**0.004**
MYC*BCL6		
Race: Black & Other vs. White	0.96 (0.65, 1.40)	0.829
Race: Asian vs. White	0.89 (0.46, 1.68)	0.735
Age	1.00 (1.00, 1.01)	0.376
MYC*IGH::*BCL2*		
Race: Black & Other vs. White	1.11 (0.72, 1.68)	0.639
Race: Asian vs. White	1.47 (0.70, 2.94)	0.292
Age	**1.01 (1.00, 1.03)**	**0.010**
BCL6*BCL2		
Race: Black & Other vs. White	1.19 (0.82, 1.74)	0.360
Race: Asian vs. White	1.42 (0.77, 2.61)	0.252
Age	**1.02 (1.01, 1.03)**	**<0.001**
BCL6*IGH::*MYC*		
Race: Black & Other vs. White	1.12 (0.75, 1.65)	0.576
Race: Asian vs. White	0.94 (0.47, 1.81)	0.865
Age	1.00 (0.99, 1.01)	0.933
BCL2*IGH::*MYC*		
Race: Black & Other vs. White	0.94 (0.62, 1.42)	0.769
Race: Asian vs. White	1.55 (0.80, 2.92)	0.182
Age	**1.01 (1.00, 1.02)**	**0.012**
Aggressive HGBCL (binary outcome) ^a^		
Race: Black & Other vs. White	1.12 (0.36, 2.96)	0.832
Race: Asian vs. White	1.26 (0.19, 4.77)	0.765
Age	**1.05 (1.02, 1.09)**	**0.004**

CI = confidence interval. ^a^ Results were calculated by logistic regression. A HGBCL was defined as at least one of (*MYC*, *IGH*::*MYC*) = 1 (abnormal), AND at least one of (*BCL2*, *IGH*::*BCL2*) = 1 (abnormal). Bold font shows either statistically significant or marginal statistically significant (0.05 < *p* < 0.10).

## Data Availability

All requests for primary data and experimental reagents should be addressed to yihuang@umbc.edu and yzou19@jhmi.edu.

## Supplementary Materials

The following supporting information can be downloaded at https://www.mdpi.com/article/10.3390/biomedicines13112782/s1: Table S1: Disease types and cell-of-origin subtypes in the study cohort., Table S2: Comparison of age, sex, and genetic biomarkers across analytic sample vs. the sample excluded due to missing race., Table S3: Race differences in gene abnormalities with adjustments for confounders., Table S4: Racial differences in gene–gene interaction and HGBCL with adjustments for confounders., Table S5: Race Differences (White and Non-white) in Gene–gene interactions.

## Abbreviations

The following abbreviations are used in this manuscript:

DLBCL	Diffuse large B-cell lymphoma
FISH	fluorescence in situ hybridization
HGBCL	high-grade B-cell lymphoma
IGH	immunoglobulin heavy chain
Non-IGs	non-immunoglobulin partner genes
