# Supplementary material for: Racial Differences in the Molecular Genetic Biomarkers of Diffuse Large B-Cell Lymphoma
Source: Biomedicines. 2025 Nov 14;13(11):2782. doi: 10.3390/biomedicines13112782 (PMC12650435; doi:10.3390/biomedicines13112782)
Supplement: Supplementary file 1 [file biomedicines-13-02782-s001.zip › biomedicines-3934905-supplementary.docx]

**Supplementary Materials**

**Table S1.** **Disease types and cell-of-origin subtypes in the study cohort.**

| Disease types | Overall (n=919) | White (n=661) | Black (n=157) | Asian (n=57) | Other (n=44) |
| --- | --- | --- | --- | --- | --- |
| *de novo* | 758 (82.48) | 538 (81.39) | 134 (85.35) | 48 (84.21) | 38 (86.35) |
| Transformed | 107 (11.64) | 83 (12.56) | 15 (9.55) | 7 (12.28) | 2 (4.55) |
| NA | 54 (5.88) | 40 (6.05) | 8 (5.10) | 2 (3.51) | 4 (9.09) |
|  |  |  |  |  |  |
| Transformed from | Overall (n=919) | White (n=661) | Black (n=157) | Asian (n=57) | Other (n=44) |
| FL | 67 (7.29) | 54 (8.17) | 8 (5.10) | 5 (8.77) | 0 (0.00) |
| MZL | 18 (1.96) | 10 (1.51) | 5 (3.18) | 1 (1.75) | 2 (4.55) |
| CLL/SLL | 12 (1.31) | 10 (1.51) | 2 (1.27) | 0 (0.00) | 0 (0.00) |
| Other | 10 (1.09) | 9 (1.36) | 0 (0.00) | 1 (1.75) | 0 (0.00) |
|  |  |  |  |  |  |
| Cell-of-origin subtypes | Overall (n=919) | White (n=661) | Black  (n=157) | Asian (n=57) | Other (n=44) |
| GCB | 452 (49.18) | 322 (48.71) | 79 (50.32) | 32 (56.14) | 19 (43.18) |
| Non-GCB | 281 (30.58) | 200 (30.26) | 47 (29.94) | 18 (31.58) | 16 (36.36) |
| ABC | 67 (7.29) | 50 (7.56) | 12 (7.64) | 4 (7.02) | 1 (2.27) |
| Unclassified | 32 (3.48) | 23 (3.48) | 7 (4.46) | 0 (0.00) | 2 (4.55) |
| NA | 87 (9.47) | 66 (9.98) | 12 (7.64) | 3 (5.26) | 6 (13.64) |

All values are presented as n (%). ABC: activated B-cell; CLL: Chronic lymphocytic leukemia;

FL: Follicular lymphoma; GCB: germinal center B-cell; MZL: marginal zone lymphoma; NA: not available;

non-GCB: non-germinal center B-cell; Other: including lymphoplasmacytic lymphoma, Waldenström macroglobulinemia, mantle cell lymphoma, nodal B-cell NOS, low-grade B-cell lymphoma, etc.;

SLL: Small lymphocytic lymphoma. All P-values > 0.05 (a Chi^2^ test).

**Table S2.** **Comparison of age, sex, and genetic biomarkers across analytic sample vs. the sample excluded due to missing race.**

| **Variable** | **Overall** | **Sample**  **size** | **Analytic Sample** | **Exclusion**  **(no Race)** |  |
| --- | --- | --- | --- | --- | --- |
|  | (n=1,167) | (available) | (n= 919) | (n=248) | **P-value** |
| Age: mean (SD*) | 63.74 (16.93) | 1166 | 63.58 (17.11) | 64.32 (16.26) | 0.53 |
| Sex (female) | 478 (41.03%) | 1165 | 369 (40.15%) | 109 (44.31%) | 0.27 |
| *MYC* | 177 (17.40%) | 1017 | 145 (17.88%) | 32 (15.53%) | 0.49 |
| *BCL6* | 208 (24.10%) | 863 | 173 (25.22%) | 35 (19.77%) | 0.16 |
| *BCL2* | 217 (24.58%) | 883 | 187 (26.56%) | 30 (16.76%) | **0.01** |
| *IGH*::*BCL2* | 150 (18.07%) | 830 | 124 (18.87%) | 26 (15.03%) | 0.29 |
| *IGH*::*MYC* | 89 (8.94%) | 996 | 77 (9.69%) | 12 (5.97%) | 0.13 |

SD*: standard deviation

| **Table S3: Race differences in gene abnormalities with adjustments for confounders.** | | | |
| --- | --- | --- | --- |
|  |  | **Logistic Regression** | |
| **Variables** | | **Odds Ratio (95% CI)** | **P-value** |
| *MYC* | |  |  |
| *Race: Black vs. White* | | 0.72 (0.43, 1.19) | 0.216 |
| *Asian vs. White* | | 0.73 (0.31, 1.50) | 0.421 |
| *Other vs. White* | | 0.46 (0.13, 1.19) | 0.151 |
| *Age* | | 1.00 (0.99, 1.01) | 0.572 |
| *BCL6* | |  |  |
| *Race: Black vs. White* | | 1.22 (0.76, 1.94) | 0.402 |
| *Asian vs. White* | | 1.00 (0.47, 1.99) | 0.998 |
| *Other vs. White* | | 1.27 (0.56, 2.65) | 0.544 |
| *Age* | | **1.01 (1.00, 1.02)** | **0.047** |
| *BCL2* | |  |  |
| *Race: Black vs. White* | | 0.92 (0.56, 1.48) | 0.743 |
| *Asian vs. White* | | 1.69 (0.87, 3.21) | 0.112 |
| *Other vs. White* | | 1.22 (0.54, 2.54) | 0.615 |
| *Age* | | **1.03 (1.01, 1.04)** | **0.000** |
| *IGH*::*BCL2* | |  |  |
| *Race: Black vs. White* | | 1.18 (0.68, 1.98) | 0.542 |
| *Asian vs. White* | | **1.90 (0.86, 3.96)** | **0.095** |
| *Other vs. White* | | 1.51 (0.58, 3.5) | 0.362 |
| *Age* | | **1.03 (1.01, 1.04)** | **0.000** |
| *IGH*::*MYC* | |  |  |
| *Race: Black vs. White* | | 0.87 (0.44, 1.60) | 0.680 |
| *Asian vs. White* | | 0.55 (0.13, 1.58) | 0.337 |
| *Other vs. White* | | 0.61 (0.14, 1.81) | 0.432 |
| *Age* | | **0.98 (0.97, 1.00)** | **0.005** |

| **Table S4: Racial differences in gene-gene interaction and HGBCL with adjustments for confounders.** | | | | |
| --- | --- | --- | --- | --- |
|  |  | Proportional odds model  (Accounting ordering in outcome) | |  |
| **Variables** | | Regression Coefficient (95% CI) | P-value |  |
| ***MYC***IGH*::*MYC*** | |  |  |  |
| *Race: Black vs. White* | | 0.71 (0.42, 1.18) | 0.205 |  |
| *Asian vs. White* | | 0.64 (0.26, 1.38) | 0.293 |  |
| *Other vs. White* | | 0.49 (0.14, 1.28) | 0.191 |  |
| *Age* | | 1.00 (0.99, 1.01) | 0.401 |  |
| ***IGH*::*MYC***IGH*::*BCL2*** | |  |  |  |
| *Race: Black vs. White* | | 1.41 (0.87, 2.26) | 0.157 |  |
| *Asian vs. White* | | **1.92 (0.91, 3.89)** | **0.076** |  |
| *Other vs. White* | | 1.06 (0.41, 2.44) | 0.897 |  |
| *Age* | | **1.01 (1.00, 1.02)** | **0.060** |  |
| ***BCL2***IGH*::*BCL2*** | |  |  |  |
| *Race: Black vs. White* | | 1.17 (0.67, 2.00) | 0.562 |  |
| *Asian vs. White* | | **2.11 (1.00, 4.38)** | **0.049** |  |
| *Other vs. White* | | 1.51 (0.57, 3.51) | 0.368 |  |
| *Age* | | **1.03 (1.01, 1.04)** | **0.000** |  |
| ***BCL6***IGH*::*BCL2*** | |  |  |  |
| *Race: Black vs. White* | | 1.3 (0.82, 2.05) | 0.258 |  |
| *Asian vs. White* | | **1.78 (0.90, 3.5)** | **0.096** |  |
| *Other vs. White* | | 1.18 (0.53, 2.52) | 0.673 |  |
| *Age* | | **1.02 (1.01, 1.03)** | **0.000** |  |
| ***MYC***BCL2*** | |  |  |  |
| *Race: Black vs. White* | | 0.92 (0.59, 1.41) | 0.705 |  |
| *Asian vs. White* | | 1.33 (0.7, 2.46) | 0.377 |  |
| *Other vs. White* | | 0.61 (0.27, 1.29) | 0.219 |  |
| *Age* | | **1.01 (1.01, 1.02)** | **0.004** |  |
| ***MYC***BCL6*** | |  |  |  |
| *Race: Black vs. White* | | 1.03 (0.67, 1.56) | 0.893 |  |
| *Asian vs. White* | | 0.90 (0.46, 1.68) | 0.736 |  |
| *Other vs. White* | | 0.78 (0.38, 1.52) | 0.472 |  |
| *Age* | | 1.00 (1.00, 1.01) | 0.404 |  |
| ***MYC***IGH*::*BCL2*** | |  |  |  |
| *Race: Black vs. White* | | 1.20 (0.76, 1.89) | 0.427 |  |
| *Asian vs. White* | | 1.47 (0.70, 2.94) | 0.292 |  |
| *Other vs. White* | | 0.79 (0.31, 1.80) | 0.599 |  |
| *Age* | | **1.01 (1.00, 1.03)** | **0.013** |  |
| ***BCL6***BCL2*** | |  |  |  |
| *Race: Black vs. White* | | 1.16 (0.76, 1.76) | 0.492 |  |
| *Asian vs. White* | | 1.42 (0.77, 2.61) | 0.252 |  |
| *Other vs. White* | | 1.30 (0.65, 2.57) | 0.451 |  |
| *Age* | | **1.02 (1.01, 1.03)** | **0.000** |  |
| ***BCL6***IGH*::*MYC*** | |  |  |  |
| *Race: Black vs. White* | | 1.12 (0.72, 1.73) | 0.602 |  |
| *Asian vs. White* | | 0.94 (0.47, 1.81) | 0.865 |  |
| *Other vs. White* | | 1.10 (0.53, 2.22) | 0.786 |  |
| *Age* | | 1.00 (0.99, 1.01) | 0.930 |  |
| ***BCL2***IGH*::*MYC*** | |  |  |  |
| *Race: Black vs. White* | | 0.99 (0.62, 1.55) | 0.962 |  |
| *Asian vs. White* | | 1.55 (0.8, 2.92) | 0.182 |  |
| *Other vs. White* | | 0.78 (0.32, 1.71) | 0.553 |  |
| *Age* | | **1.01 (1.00, 1.02)** | **0.013** |  |
| **Aggressive HGBCL**  (binary outcome) | | Logistic Regression |  |  |
| *Race: Black vs. White* | | 1.12 (0.31, 3.17) | 0.842 |  |
| *Asian vs. White* | | 1.26 (0.19, 4.77) | 0.765 |  |
| *Other vs. White* | | 1.11 (0.06, 6.01) | 0.922 |  |
| *Age* | | **1.05 (1.02, 1.09)** | **0.004** |  |
|  | |  |  |  |

**Table S5. Race Differences (White and Non-white) in Gene*gene interactions**

|  | **Overall** | | | | **White** | | | | **Non-White** | | | |  |
| --- | --- | --- | --- | --- | --- | --- | --- | --- | --- | --- | --- | --- | --- |
|  | (n=919) | | | | (n=661) | | | | (n=258) | | | | **P-value** |
|  | 0* | 1* | 2* | NA | 0 | 1 | 2 | NA | 0 | 1 | 2 | NA |  |
| *MYC***BCL2* | 412 | 205 | 41 | 261 | 295 | 147 | 33 | 186 | 117 | 58 | 8 | 75 | 0.52 |
| *IGH*::*MYC***IGH*::*BCL2* | 452 | 145 | 4 | 318 | 328 | 95 | 3 | 235 | 124 | 50 | 1 | 83 | 0.13 |
| *MYC***BCL6* | 399 | 233 | 34 | 253 | 287 | 166 | 28 | 180 | 112 | 67 | 6 | 73 | 0.66 |
| *MYC***IGH*::*MYC* | 645 | 72 | 69 | 133 | 454 | 56 | 54 | 97 | 191 | 16 | 15 | 36 | **0.05** |
| *MYC***IGH*::*BCL2* | 425 | 150 | 25 | 319 | 304 | 102 | 19 | 236 | 121 | 48 | 6 | 83 | 0.63 |
| *BCL6***BCL2* | 367 | 249 | 40 | 263 | 269 | 175 | 28 | 189 | 98 | 74 | 12 | 74 | 0.39 |
| *BCL6***IGH*::*BCL2* | 355 | 195 | 18 | 351 | 258 | 132 | 13 | 258 | 97 | 63 | 5 | 93 | 0.27 |
| *BCL6***IGH*::*MYC* | 429 | 219 | 9 | 262 | 312 | 155 | 7 | 187 | 117 | 64 | 2 | 75 | 0.68 |
| *BCL2***IGH*::*BCL2* | 465 | 19 | 104 | 331 | 337 | 13 | 70 | 241 | 128 | 6 | 34 | 90 | 0.29 |
| *BCL2***IGH*::*MYC* | 436 | 199 | 9 | 272 | 318 | 141 | 8 | 194 | 121 | 58 | 1 | 78 | 0.91 |

Gene-pair = 0*, if both genes normal (0,0). Gene-pair = 1*, if one out of the two genes abnormal (0,1) or (1,0). Gene-pair = 2*, if both genes abnormal (1,1). P-values are from Goodman-Kruskal's gamma test, accounting for the ordering of gene pair abnormality’s values (ordinal).
